# AN INTERNATIONAL PERSPECTIVE ON GOAL SETTING AND INTERDISCIPLINARY COLLABORATION WITHIN REABLEMENT PROGRAMS

**DOI:** 10.1093/geroni/igad104.3047

**Published:** 2023-12-21

**Authors:** Lise Buma, Hanne Tuntland, Sandra Zwakhalen, Silke Metzelthin

**Affiliations:** Maastricht University, Maastricht, Limburg, Netherlands; Western Norway University of Applied Sciences, Bergen, Hordaland, Norway; Maastricht University, Maastricht, Limburg, Netherlands; Maastricht University, Care and Public Health Research Institute, Maastricht, Limburg, Netherlands

## Abstract

Over the last two decades, reablement has been studied and implemented in more than 16 countries. It is a person-centred approach aiming to enhance individuals’ (physical) functioning and increase or maintain their independence in meaningful activities. Goal setting and interdisciplinary collaboration are identified as important key elements of reablement. Due to limited intervention descriptions in the scientific literature, it is not clear how these two elements are applied in practice. This hinders the uptake of reablement services among healthcare care providers on national and international level. This study aimed to provide insight into: (1) goal setting; and (2) interdisciplinary collaboration within reablement programs. Therefore, a qualitative study in Norway and the Netherlands was conducted. In both countries, reablement staff (n=14) was interviewed. Data was analysed using both deductive and inductive content analysis. To increase rigor in terms of credibility, transferability, dependability and conformability; member checking, uniform data collection methods, note-taking, and direct quotations were used to analyse and present the data in this study. These focus groups resulted in, on the one hand, a clear overview of procedures regarding goal setting, treatment and evaluation methods, and on the other hand, valuable insights into staff experiences regarding interdisciplinary collaboration. In addition, facilitators and barriers for the implementation of goal setting and interdisciplinary collaboration in reablement programs were identified. This study resulted in valuable knowledge for healthcare providers to effectively implement reablement in practice.

